# Mirtazapine-Induced Bicytopenia in a Patient With Multifactorial Delirium: A Case Report

**DOI:** 10.7759/cureus.107522

**Published:** 2026-04-22

**Authors:** Benyam G Yirdaw, Alexander Bulcock

**Affiliations:** 1 General Medicine/Acute Medicine, Fairfield General Hospital, Manchester, GBR; 2 Geriatrics, Fairfield General Hospital, Manchester, GBR

**Keywords:** agranulocytosis, bicytopenia, case report, mirtazapine, multifactorial delirium

## Abstract

Mirtazapine is often preferably used in geriatric patients with mood disorders due to its favourable side effects such as somnolence and weight gain. However, mirtazapine is associated with a rare but fatal side effect, agranulocytosis, which is not widely described. We present a case of mirtazapine-induced bicytopenia (moderate anaemia and leukopenia) in a geriatric patient. Notwithstanding scant available reports of mirtazapine-induced neutropenia, a case of iatrogenic anaemia from the drug is absent as per our literature review, although underreporting and undocumented cases are possibilities.

We report a drug-induced bicytopenia in a 71-year-old Caucasian female patient, with a background of schizoaffective disorder, following mirtazapine use. The report shows declines in cell lines, 10 days after dose titration, complicated by SARS-CoV-2 infection and fever of unknown origin, with complete resolution of bicytopenia (moderate anaemia and leukopenia) following mirtazapine cessation. This was demonstrated by the definite temporal association displayed through the standardised Naranjo Adverse Drug Reaction Probability Scale.

This case reports mirtazapine's rare side effects, anaemia and leukopenia and underscores the pharmacovigilance required in geriatric patients, who are prone to infections and complications of anaemia.

## Introduction

Mental health disorders such as depression are common in admitted geriatric patients. Antidepressants are prescribed to patients with moderate and severe depression [1,2]. However, prescribing in elderly patients with co-morbidities, compromised immunity, and frailty begs for pharmacovigilance to prevent fatal side effects. 

The choice of antidepressants is governed by clinical contexts of patients and patient’s preference [3]. Mirtazapine, an atypical tetracyclic antidepressant, which is often used in cases of depression complicated by anxiety or insomnia, is often used as an alternative to selective serotonin reuptake inhibitors (SSRIs), where SSRIs fail or side effects preclude use. Desirable side effects of mirtazapine such as somnolence, increased appetite and weight gain could be employed in this cohort of patients, where poor oral intake, weight loss and sleep disturbance contribute to progression of frailty [3]. However, mirtazapine has a rare but fatal side effect, agranulocytosis, which if not recognised and addressed promptly could prove fatal with neutropenic sepsis, especially in frail elderly patients, who are often immunocompromised [4].

There are limited reports and writings on this side effect so far. Premarketing trials showed a crude incidence of mirtazapine-induced severe neutropenia (absolute neutrophil count (ANC) <500 /mm^3^) of 1.1 per 1000 patients [5]. A handful of case reports described mirtazapine-induced neutropenia, thrombocytopenia and bicytopenia [4-10]. However, iatrogenic anaemia from mirtazapine is absent in the literature. We present a case of mirtazapine-induced bicytopenia (moderate anaemia and leukopenia) in a patient admitted with multifactorial delirium on a background of schizoaffective disorder.

This case is important in that it reports mirtazapine's rare and fatal side effects, agranulocytosis and anaemia and includes complications in a geriatric patient such as COVID-19 (coronavirus disease 2019) and fever of unknown origin. Moreover, it emphasises the pharmacovigilance required in this cohort with a recommendation to prevent complications. 

## Case presentation

This is a 71-year-old Caucasian female patient, who presented to the emergency department in October 2025, with unusual behaviour, agitation and paranoia of two weeks duration, which was worsened in the few days prior to admission. The patient had a recent hospital admission, where, on discharge, chlorpromazine was stopped due to drowsiness. They have a past medical history of schizoaffective disorder, for which they were on sertraline, chlorpromazine and aripiprazole. Other past medical history includes stage 4 chronic kidney disease (CKD) and hypertension and was on ferrous fumarate, oxybutynin and procyclidine as regular medications. On examination, the patient was unkempt, confused and agitated. Neurologic exam revealed a Glasgow Coma Scale (GCS) of 13/15 and marked agitation, which made further examination difficult.

On investigation, blood tests were marked for a disproportionately raised alkaline phosphatase 282 u/L (reference range: 30-130 u/L) with fairly normal blood tests, haemoglobin level 116 g/L (reference range: 115-165 g/L), white cell count 6.6x10*9/L (reference range: 4.00-11.00x10*9) with a neutrophil count of 4.2x10*9/L (reference range: 2.5-7.5x10*9), platelet count 179x10*9/L (reference range: 150-450x10*9/L), bilirubin 5 umol/L (reference range: 0-20 umol/L), and alanine transaminase 30 u/L (reference range: 7-40 u/L) and chronically deranged renal function consistent with CKD. Thyroid-stimulating hormone, serum iron and vitamin B12 and folate were in the normal range. Abdominal ultrasound suggested mild cholecystitis with cholelithiasis.

The patient was diagnosed with delirium caused by multiple factors such as cholecystitis, inadvertently missed chlorpromazine doses and increased anticholinergic burden from medications such as oxybutynin and procyclidine. The patient was started on amoxicillin/clavulanic acid, oxybutynin was switched to mirabegron, and the dose of procyclidine was reduced.

The patient's symptoms worsened on arrival at the geriatric medicine ward. She became more agitated and developed paranoia to the point where deprivation of liberty safeguards (DOLS) were put in place. Following mental health assessment, the patient was started on 15 mg of mirtazapine, which was increased to 30 mg nocte after a week's time. The patient was also started on trazodone 50 mg, given the lack of improvement in the following week.

After three weeks of combined antipsychotic treatment, the patient improved, and the delirium subsided. However, the patient developed fever, cough and feeling generally unwell and scored 2 on early warning score for temperature 39.3 °C, at which point, investigations with bloods, chest X-ray and swabs were done. Blood tests showed haemoglobin 84 g/L, white cell count 3.2 x10*9/L with neutrophil count of 2.3x10*9/L and platelet count - 178x10*9/L with C-reactive protein (CRP) 56.1 mg/L. SARS-CoV-2 (severe acute respiratory syndrome coronavirus 2) nasal swab test came up as positive.

With the impression of bicytopenia (moderate anaemia and leukopenia) secondary to mirtazapine and SARS-CoV-2 infection, the patient was isolated, mirtazapine was stopped, and trazodone was increased while keeping the rest of the antipsychotic and depression medications. 

Despite 10 days of isolation, the patient manifested swinging persistent fever, for which she was screened with full blood count, C-reactive protein, blood culture, chest x-ray, HIV screening test, beta D-glucan antifungal test, immunology for vasculitis and echocardiography, all of which came back negative and gave a picture of fever of unknown origin.

After 20 days of mirtazapine cessation, the cell lines improved progressively to baseline with haemoglobin 106 g/L, white cell count 9.1x10*9/L with a neutrophil count of 6.5x10*9/L and platelet count 237x10*9/L.

The fever subsided on its own without further intervention; the patient became medically fit and passed physiotherapy assessments for discharge and was back to baseline mobility wise. Finally, the patient was discharged to intermediate care, to wait instalment of package of care.

On review, three months later in March 2026, at which point the patient was readmitted for worsening of the schizoaffective disorder, blood tests revealed a sustained improvement in cell lines throughout admission. A full blood count in March during this admission revealed a haemoglobin level of 103 g/L, a white cell count of 5.3x10*9/L with neutrophil count 3.8x10*9/L and a platelet count of 203x10*9/L. Blood levels remained stable, and the patient was discharged by the end of March. 

**Figure 1:**
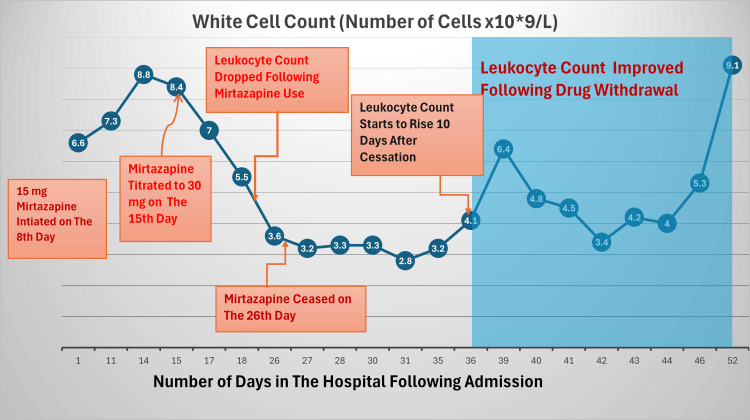
Pattern of White Cell Count in Relation to Mirtazapine Use Over the Course of Admission Period This line chart relates the number of days in the hospital to white cell count and portrays how the number of white blood cells dipped following mirtazapine use, with improvement after drug cessation.

Figure 1 shows patterns of white cell count changes following mirtazapine initiation and discontinuation.

Table 1 presents a tabulated version of Figure 1 detailing the number of white cells for the respective days in the hospital.

**Table 1:** Pattern of White Cell Count in Relation to Mirtazapine Use Over the Course of Admission Period This table presents the number of white cells per litre on the described days from admission. Reference range: 4.0-11.0x10*9/L

Number of Days From Admission	White Cell Count (Number of Cells x 10*9/L)
1	6.6x10*9/L
11	7.3x10*9/L
14	8.8x10*9/L
15	8.4x10*9/L
17	7x10*9/L
18	5.5x10*9/L
26	3.6x10*9/L
27	3.2x10*9/L
28	3.3x10*9/L
30	3.3x10*9/L
31	2.8x10*9/L
35	3.2x10*9/L
36	4.1x10*9/L
39	6.4x10*9/L
40	4.8x10*9/L
41	4.5x10*9/L
42	3.4x10*9/L
43	4.2x10*9/L
44	4x10*9/L
46	5.3x10*9/L
52	9.1x10*9/L

**Figure 2:**
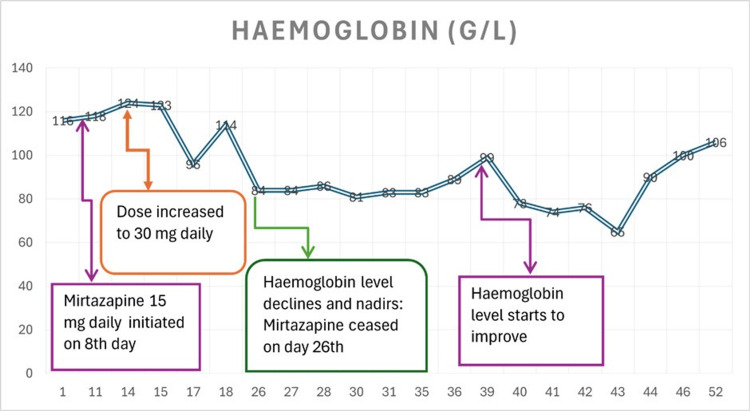
Haemoglobin Level Mapped to Mirtazapine Use Over the Course of Days of Hospital Admission The level of haemoglobin in G/L* is represented on the Y-axis and related to the number of days in the hospital. Haemoglobin level declined following mirtazapine use and nadired at 81 G/L and improved after drug withdrawal. * G/L: gram per litre

The graph displayed in Figure 2 maps haemoglobin level changes following mirtazapine use.

 In Table 2, the tabulation of the haemoglobin level of the patient is presented as measured throughout the current admission. 

**Table 2:** Haemoglobin Level in Relation to Mirtazapine Use Over the Course of Days of Hospital Admission The level of haemoglobin as measured in gram per litre is presented for the respective days from admission. Reference range: 115 – 165 G/L

Number of Days From Admission	Haemoglobin (G/L)
1	116 G/L
11	118 G/L
14	124 G/L
15	123 G/L
17	96 G/L
18	114 G/L
26	84 G/L
27	84 G/L
28	86 G/L
30	81 G/L
31	83 G/L
35	83 G/L
36	89 G/L
39	99 G/L
40	78 G/L
41	74 G/L
42	76 G/L
43	65 G/L
44	90 G/L
46	100 G/L
52	106 G/L

The main differential diagnoses considered but ruled out as possible causes of the bicytopenia are nutritional deficiencies, iron deficiency and malignancy. The patient had been followed and managed by dieticians in the ward for over a month and vitamin B12 and folate were in the normal range, making nutritional causes unlikely.

Serum iron and transferrin saturation on admission were normal despite a one-off record of low iron at a point in the hospital stay; however, the close temporal association between mirtazapine use and withdrawal and cell line changes and therapeutic dose ferrous fumarate for over two months makes iron deficiency less likely as a culprit, although its contributory role can be debated.

Lack of clinical and imaging findings, the improvement of cell lines without oncologic intervention and the close temporal association with mirtazapine ruled out malignancy as a cause.

CKD is a common cause of anaemia through erythropoietin and iron deficiency among other possible less common mechanisms. However, the close temporal relation between mirtazapine use and haemoglobin decline from baseline, and drug withdrawal and haemoglobin rise without any erythropoietin-stimulating agent, the microcytic phenotype of the anaemia and normal levels of serum iron, ferritin and transferrin saturation rule out CKD as the main aetiology although its contributory role cannot be disputed. 

Two confounding factors to consider are SARS-CoV-2 infection and polypharmacy. Although COVID-19 causes haematologic abnormalities such as lymphocytosis and thrombocytopenia, neutropenia is rare, which could occur in the acute infection phase or later [11]. However, the presence of a down-trending pattern of cell lines before symptoms or the diagnosis of COVID-19 and a Naranjo adverse drug reaction probability scale score of 9 excludes SARS-CoV-2 infection as a culprit.

Notwithstanding the patient had been on multiple medications including aripiprazole, which in rare cases could cause neutropenia, the fact that the patient had been on those medications for months, some of which were deprescribed, reduced (procyclidine) and switched to safer options (oxybutynin to mirabegron) makes polypharmacy an unlikely cause. Moreover, the bicytopenia followed by mirtazapine initiation and titration is a recognised response and was confirmed by the improvement of cell lines after drug withdrawal, giving a Naranjo adverse drug reaction probability scale score of 9, making the diagnosis of mirtazapine-induced drug reaction definite.

## Discussion

Premarketing trials revealed mirtazapine-induced agranulocytosis, between 9 and 61 days of initiation, with a crude incidence of severe neutropenia (ANC <500 /mm^3^) of 1.1 per 1000 patients [5]. The mechanism by which mirtazapine affects cell lines is not well described; however, one study reported mirtazapine-induced immune-mediated thrombocytopenia, where the team identified drug-induced auto-antibodies that targeted the glycoprotein complex GP IIb/IIIa on platelets [6].

There are several case reports, which documented mirtazapine-induced neutropenia in patients who were started on the drug between 10 days and 3 months [4,7-10]. Close temporal association was described with prompt resolution upon drug withdrawal [7-10]. 

One report presented a 91-year-old patient, who came to the hospital after a fall and prolonged lie, admitted with AKI and treated with intravenous fluid [7]. Blood tests showed an incidental low absolute neutrophil count on the 13th day of admission, which nadired on the 17th day to an undetectable level. The patient developed neutropenic sepsis, which necessitated intravenous antibiotics and granulocyte-colony stimulating factor. Medication review revealed that 15 mg mirtazapine was initiated three weeks before admission. The drug was ceased, and the absolute neutrophil count improved on the 21st day of admission, which remained in range six months after discharge.

In our case, 15 mg of mirtazapine was initiated on the eighth day of admission, which was titrated to 30 mg on the 15th day, resulting in a decline of the white cell count to 3.6x10*9/L on the 26th day, at which point mirtazapine was ceased. The leukocyte count further deteriorated to 2.8x10*9/L, complicated by COVID-19, followed by fever of unknown origin. Ten days after drug withdrawal, the white cell count improved to 4.1x10*9/L and remained in range two weeks later, where it was last checked before discharge.

Both reports show neutropenia following recent mirtazapine initiation. However, in our case, the neutropenia developed upon titration of the drug to 30 mg, while in the above following initiation of 15 mg of mirtazapine three weeks prior to presentation [7]. Moreover, this report includes an additional haematologic abnormality, moderate anaemia. Cell lines improved in both cases following drug withdrawal. 

Another case report reported a 72-year-old patient who presented with fever and epistaxis [10]. Blood tests revealed white blood cell, neutrophil, and platelet counts of 3.27 x 10*9/L, 0.17 x 10*9/L, and 28.10 x 10*9/L, respectively. Drug review showed initiation of 15 mg of mirtazapine for low mood and alysosis 10 days prior to presentation. Possible differentials were ruled out with blood tests including haematinics, immunology tests and infectious screen. Bone marrow biopsy demonstrated hypocellularity with differentiation of the myeloid lineage and mildly decreased megakaryocytes. Mirtazapine was ceased and escitalopram and alprazolam were subsequently started. Platelet count raised to 89x10*9/L and 100x10*9/L on the seventh day and 14th day after drug withdrawal, respectively. Blood tests 21 days after mirtazapine cessation revealed an absolute neutrophil count of 2.5x10*9/L and a platelet count of 219x10*9/L. 

In line with the current report, the above case showcases acute changes in cell lines following mirtazapine initiation and a clear temporal association with the return of the cell line to baseline following drug withdrawal in 21 days [10]. However, it rather demonstrated severe thrombocytopenia as opposed to the moderate anaemia in the current report. In contrast to our case, the above report presented cell line changes in association with a regimen of 15 mg daily.

A similar report described a case of mirtazapine-induced neutropenia in a 93-year-old dementia patient, who was started on 15 mg of mirtazapine for sustained depressive symptoms in a geriatrics clinic [8]. Baseline white cell count was 7.8x10*9/L with 41% neutrophil (ANC = 3.198x10*9/L). Five months later, the dose was titrated to 30 mg for persistent low mood. Blood tests, at this level, showed 8.9x10*9/L, with 38% neutrophil (ANC: 3.382x10*9/L). On follow-up, three months later, neutrophil count declined to 34% of the white cell count (8.02x10*9/L) with ANC of 2.727x10*9/L. Mirtazapine was swapped for sertraline 50 mg. Two months after drug withdrawal, blood tests revealed a total WBC count of 9.240x10*9/L, with 39% neutrophil (ANC: 3.604x10*9/L).

The above report demonstrated a more protracted, nevertheless, a temporal relation between mirtazapine use and neutrophil decline [8]. The neutrophil count change was noted three months after mirtazapine titration to 30 mg, which contrasts with unaltered cell lines on the 15 mg regimen. Our case shows a more acute change on a 30 mg regimen with a prompt response following drug withdrawal. A possible reason for the above could be longer follow-up dates, although this can't, at this point, be ascertained without available tests, given the incidental nature of the finding.

The current report reviewed similar reports, discussed and ruled out possible common differentials and used an objective method to define a mirtazapine related adverse drug reaction using the Naranjo adverse drug reaction probability scale. However, this report does not include specialised investigations such as bone marrow biopsy and does not describe mechanisms. 

We haven’t yet found a case of anaemia caused by mirtazapine use, making this case report peculiar in that a close temporal association has been made between mirtazapine use and bicytopenia (moderate anaemia and leukopenia). We, however, recognise possibilities of under-reporting or undocumented cases.

In our case, the patient developed moderate anaemia and leukopenia after 10 days of drug titration, and cell lines showed improvement after a week of drug withdrawal, with a return to baseline after 20 days.

The patient developed COVID and a fever of unknown origin, which subsided with drug withdrawal. However, there is a case report of fatal septic shock due to mirtazapine-induced leukopenia [4].

## Conclusions

Mirtazapine is often used as an alternative to SSRIs in geriatric patients with depression. Notwithstanding its effectiveness and desirable side effects such as somnolence and weight gain, mirtazapine can cause a rare but potentially fatal leukopenia, which is particularly important in this cohort of patients, who are prone to immunosuppression in the context of multiple co-morbidities. Our case reports dangerous side effects of mirtazapine, moderate anaemia and leukopenia. Given the predisposition of geriatric patients to infection, we suggest close monitoring of cell lines for patients started on mirtazapine and prompt drug withdrawal should cell lines are affected.
